# American Medical Association

**Published:** 1899-04

**Authors:** G. V. I. Brown, Eugene S. Talbot

**Affiliations:** Chairman; Secretary


### AMERICAN MEDICAL ASSOCIATION.

To the Editor:

Sir,—The next meeting of the American Medical Association will be held in Columbus, Ohio, June 6 to 9, 1899.

The Section on Stomatology will be of unusual interest, since its programme will be unlike any ever presented at any other dental society meeting. It will be made up mostly, if not entirely, of original papers, although the papers presented before this section have always been of high order. Papers upon the manipulation and technique of operations and mechanical dentistry are never read at these meetings. Only such subjects as are of interest to the dental and medical professions are received. These papers have always been selected by the secretary with the greatest care. There is no business to transact in the section. Everything in this matter is referred to the Business Committee (composed of the last three retired chairmen of sections), which meets every afternoon at five P.M. to consider the welfare of the whole Association and of each section. These matters are discussed, and, if favorably considered, they are reported favorably to the general meeting. This method relieves the sections and general meetings of considerable routine and unnecessary work, and also gives more time to do the regular business.

On Tuesday afternoon a committee of three is appointed in each section to nominate officers, a chairman and secretary for the following year. The committee reports Wednesday, at which time the candidates are elected. Since the offices of chairman and secretary carry no great honor, there is very little desire on the part of the members for the position, hence politics never mar the session.

With the exception of a few years, the present secretary has been in office continuously since the section was established, eighteen years ago. There are many advantages in having a permanent secretary, since a new chairman is elected nearly every year; a permanent secretary is familiar with the methods of conducting the section, and knows just whom to call on for papers, no matter in what particular part of the country the Association may meet. The character of the papers are always under his supervision. With his general knowledge of the workings of the general meetings, as well as the section, if he is interested the work goes on without friction.

The meetings of the Section on Stomatology are never largely attended, principally for the reason that there are no attractions except the advancement of dental science. The smallest attendance at any one session was twelve; the largest was ninety-six, with an average of twenty-eight.

The members of the Section on Stomatology have never encouraged or desired numbers. They have always felt that an attendance of even ten or twelve who were good thinkers and talkers and of sufficient intelligence to discuss the various papers presented was much more to be desired than a large number of uninterested people.

Dentists are admitted in the same manner as physicians. Dentists holding the D.D.S. degree, upon presenting credentials from their State or local society and the payment of five dollars, can become members of the Association, which also entitles them to the Journal of the Association for the coming year.

Any one who wishes to attend the sessions of the Section on Stomatology is permitted to do so.

The following list is the preliminary programme of the meeting to date:

Chairman's Address, Dr. G. V. I. Brown, Milwaukee.

"Actinomycosis," Dr. Ludwig Hektoen, Chicago.

"Evolution of Decay" (further experiments), Dr. A. C. Hart, San Francisco.

"Cocaine and Eucaine; their Relative Toxicity" (the results of original investigation and experimentation upon human, as well as animals), Dr. A. H. Peck, Chicago.

"Epithelial Structures in the Peridental Membrane" (original investigation), Dr. Frederick Noyes, Chicago.

"Infectious Ulcerative Stomatitis," Dr. John Marshall, Chicago.

"Oral Surgical Operation" (with illustrations showing remarkable results), Dr. G. V. I. Brown, Milwaukee.

"Some Points on the Etiology, Pathology, and Treatment of Persistent Pyorrhœa Alveolaris," Dr. G. T. Carpenter, Chicago.

"Interstitial Gingivitis" (so-called pyorrhœa alveolaris), giving the result of original work with large photographic illustrations showing the progress of the disease from the beginning to the exfoliation of the teeth, Dr. Eugene S. Talbot, Chicago.

"Syphilitic Infection from Dental Instruments, with Cases," Dr. W. L. Baum, Chicago.

"Professional Education and Ethics," Dr. A. E. Baldwin, Chicago.

A revised and complete programme will appear in the May number.

G. V. I. Brown, *Chairman.*
Eugene S. Talbot, *Secretary.*

